# A combination mode of climate variability responsible for extremely poor recruitment of the Japanese eel (*Anguilla japonica*)

**DOI:** 10.1038/srep44469

**Published:** 2017-03-16

**Authors:** Yong-Fu Lin, Chau-Ron Wu, Yu-San Han

**Affiliations:** 1 Department of Earth Sciences, National Taiwan Normal University, Taipei, Taiwan; 2 Institute of Fisheries Science and Department of Life Science, National Taiwan University, Taipei, Taiwan.

## Abstract

Satellite data and assimilation products are used to investigate fluctuations in the catch of Japanese eel (*Anguilla japonica*) in eastern Asian countries. It has been reported that the salinity front has extended farther south, which has shifted the eel’s spawning grounds to a lower latitude, resulting in smaller eel catches in 1983, 1992, and 1998. This study demonstrates that interannual variability in the eel catch is strongly correlated with the combination mode (C-mode), but not with the El Niño–Southern Oscillation. These eels continue to spawn within the North Equatorial Current (NEC), but the salinity front shifts south during a canonical El Niño. On the other hand, the spawning grounds accompanied by the salinity front extend farther south during the C-mode of climate variability, and eel larvae fail to join the nursery in the NEC, resulting in extremely poor recruitment in East Asia. We propose an appropriate sea surface temperature index to project Japanese eel larval catch.

The salinity front (34.5 PSU) situated between a high precipitation area (low latitude and low-salinity water) and an area with excess evaporation (high latitude and high-salinity water) in the tropical Pacific usually involves a significant atmosphere-ocean interaction. Fluctuations in the salinity front are essential to the spawning grounds of migratory fish, such as the Japanese eel (*Anguilla japonica*). These eels migrate long distances and spawn around 12–15°N to the south of the salinity front in the North Equatorial Current (NEC), and longitudinally along the western side of the West Mariana Ridge during new moon periods^1^,^2^,^3^,^4^,^5^,^6^. Meridional migration of the salinity front modulates the Japanese eel spawning grounds before the larvae join the NEC nursery, which, in turn, impacts eel abundance in East Asian countries^1^,^2^.

Previous studies have suggested that silver Japanese eels migrate seaward over thousands of kilometers to their spawning grounds south of the salinity front in the Mariana Ridge and NEC^1^,^2^,^3^,^4^,^5^,^6^ (Fig. 1). The leptocephali are capable swimmers, but their swimming speeds are slow compared with typical ocean current speeds; thus, the movement and distribution of Japanese eels are strongly affected by ocean currents during their early life stages. Newly hatched eel larvae depart from their spawning grounds, carried primarily by ocean currents, toward their growth habitats in the freshwaters of East Asia (Fig. 1). Using observations from a 52-year period (1956–2007), Shinoda *et al*. examined the distributions of larval and juvenile Japanese eels in the western North Pacific. Preleptocephali were found in the area near 142°E, 14°N to the west of the Mariana Islands from April to August. Leptocephali were widely distributed, and their size increased westward in the NEC to east of Taiwan. Metamorphosing larvae were detected east of Taiwan and the Okinawa Islands. Glass eels were found in the Kuroshio and East China Sea during winter and early spring^5^.

A previous study demonstrated that the location of the salinity front is closely related to the Southern Oscillation Index (SOI)^3^. The salinity front generally shifts to a lower latitude during El Niño, resulting in a smaller eel catch^3^. However, closer inspection indicates that the significant southward movement of the salinity front is not fully represented by the SOI. For example, three events (1982/83, 1991/92, and 1997/98) that occurred during 1980–2008 resulted in a significant southward movement of the salinity front^3^,^4^. This observation suggests that climate phenomena other than El Niño may play a role affecting the southward shift of the salinity front.

## Seasonality of the salinity front

**Figure 2:**
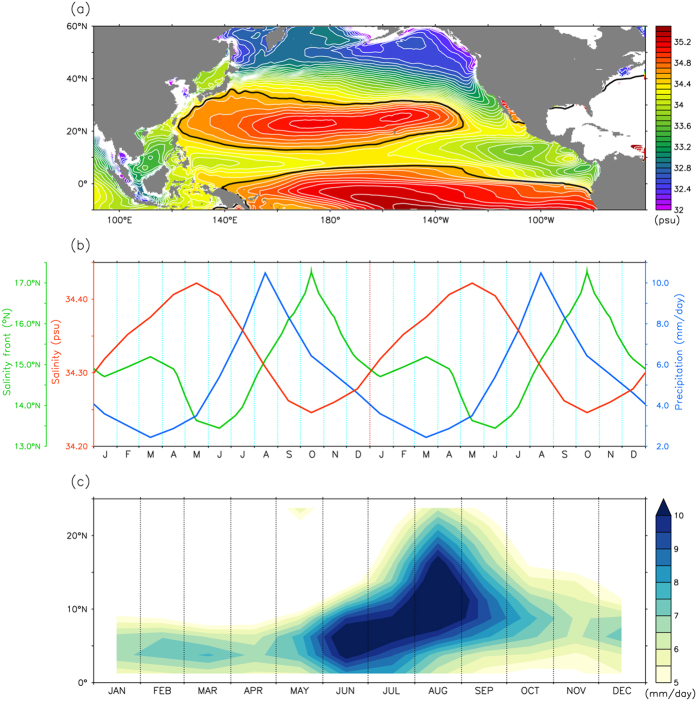
Seasonal variations of the Salinity front and ITCZ. (**a**) Annual mean of SSS averaged over depths of 0–50 m based on Simple Ocean Data Assimilation (SODA) reanalysis product. Black contours are the salinity front (34.5 PSU). Contour interval is 0.1. (**b**) Monthly climatology SSS (red curve) and precipitation fluctuations (blue curve, in mm/day) averaged over 135°–145°E and 7°–18°N, together with the position of the salinity front (green curve) averaged over 135°–145°E. (**c**) Climatological precipitation averaged over 135°–145°E.

The sea surface salinity (SSS) distribution is closely related to the precipitation pattern and significantly modulated by the Inter-tropical Convergence Zone (ITCZ)^7^. The ITCZ is the main rainfall region, and the area of fresher water (<35 PSU) just north of the equator (Fig. 2a) is a good proxy for the overlying ITCZ in the tropical Pacific^7^. The ITCZ modulates precipitation patterns throughout the tropics and strongly affects the location of the salinity front. The salinity front is generally located north of the ITCZ. On seasonal time scales, the highest salinity level occurs in May, whereas the lowest occurs in October (Fig. 2b). This seasonality is consistent with migration of the salinity front, which reaches its southernmost position in June but its northernmost in October. Changes in the SSS can indicate movement of the salinity front. When the SSS is higher (lower), the salinity front should migrate southward (northward). Furthermore, changes in rainfall are also associated with changes in SSS. The lowest precipitation amount occurs in March and the highest occurs in August, which precedes the change in SSS by ~2 months (γ = −0.9; P < 0.001).

The ITCZ is at its northernmost position in August and its southernmost in February–March (Fig. 2c), which agrees well with previous findings^8^,^9^. The sequence shows that the ITCZ migrates southward (northward), and that the high salinity waters in the mid-latitudes extend to a lower (higher) latitude, resulting in southward (northward) movement of the salinity front.

### Effect of the ITCZ on the Japanese eel spawning grounds

**Figure 3:**
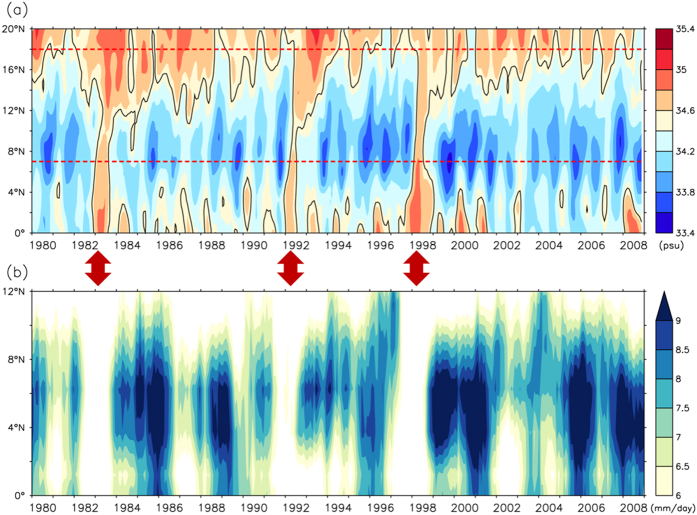
Interannual variability of the SSS and ITCZ. (**a**) Time series of monthly modeled SSS in the region of 0°–20°N, averaged over 135°–145°E with 5-month running mean. Colored shading is the same as Fig. 1a. Contours denote the salinity front, and red dash lines denote position of mean NEC (7–18°N). (**b**) Same as Fig. 2a, but for monthly precipitation with 13-month running mean. Red arrows indicate extremely southward movement events of the salinity front.

Significant interannual variation in the SSS also occurs. Figure 3a is a time series of the modeled SSS in the 0–20°N region, averaged over 135–145°E. Red lines indicate the zonal band between 7°N and 18°N, which denotes the mean position of the NEC. The variability in the salinity front generally agrees with that found in observations^3^. The salinity front moved farther south in 1983, 1992, and 1998. These three extreme events corroborate with those derived from observed salinity data measured by the Japan Meteorological Agency^3^. Among these extremely southward events, the eels spawn beyond the range of the NEC, and their larvae fail to join the NEC nursery and enter downstream of the Kuroshio, which impacts eel abundance in East Asian countries. Actual eel recruitment data confirms a significant low eel catch during 1997/98 El Niño (Fig. S1). Tracer experiments based on an observation-validated, three-dimensional model of the North Pacific Ocean circulation also verify that the latitudinal shift of spawning locations can significantly affect the recruitment success for *A. japonica*, and poor recruitment of the glass eel during extreme year (Fig. S2). Although the salinity front rarely extends to an extreme southward location, it exerts a great impact on eel catch and deserves further clarification.

Figure 3b is a time series of monthly precipitation levels, and a 13-month running mean has been applied to make the interannual variability easily discernible. The year-to-year variation was significant, and dramatically reduced precipitation occurred during the winter (December–February) and spring (March–May) of 1982/83, 1991/92, and 1997/98. The peak-to-peak comparison between the salinity front and precipitation level shows close agreement (Fig. 3a and b). Variations in precipitation are particularly induced by the ITCZ variability, and it is demonstrated that the ITCZ often shifts southward during an El Niño^10^,^11^. However, among El Niño events, intensely reduced precipitation occurred only in 1982/83, 1991/92, and 1997/98. The fact indicates that El Niño may not be solely responsible for the salinity front variability and precipitation anomaly.

**Figure 4:**
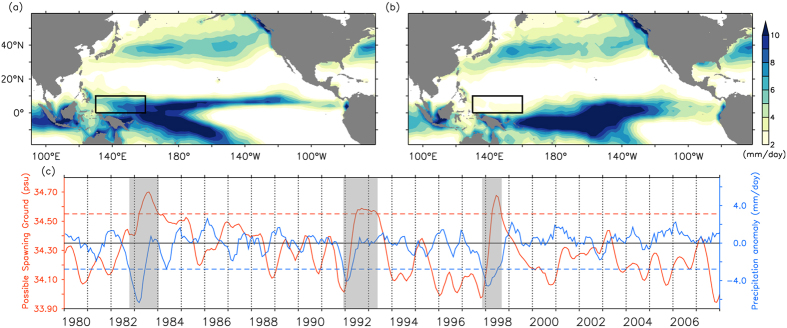
ITCZ influence on the possible spawning ground of the Japanese eel. Comparison of wintertime (DJF) precipitation composite averaged over (**a**) The canonical El Niño events, and (**b**) the three extreme events (1982/83, 1991/92 and 1997/98) during 1979–2007. (**c**) Variability of the salinity front (the indicator for the possible spawning ground), and precipitation anomaly with 5-month running mean. Precipitation anomaly is averaged over the black frame (P-BOX) in (**a,b**). Black solid line shows mean position of the salinity front. Red dashed line denotes the +1.5 standard deviation (s.d., annual mean = 34.35 PSU, s.d. = 0.14 PSU) of the salinity front variability, while blue dashed line denotes the −2 s.d. (s.d. = 1.4 mm/day) of precipitation anomaly. Gray shadings indicate the three extremely southward movement events.

Figure 4a and b respectively shows a comparison of the winter precipitation composite averaged over the canonical El Niño events and the three extreme events (1982/83, 1991/92, and 1997/98) during 1979–2007. The rainfall zone (or the ITCZ) in Fig. 4b is farther south compared with that in Fig. 4a, particularly in the northwestern tropical Pacific where potential Japanese eel spawning grounds are located. During these extreme events, the ITCZ moves significantly southward and may cross the equator, which leads to dramatically reduced precipitation in the northwestern Pacific, and the salinity front can then extend south of the equator (Fig. 3a). Eel larvae are unable to be transported into the NEC–Kuroshio system during an extreme event, because the spawning grounds have been shifted farther south. Comparison of Fig. 4a and b indicates that southward migration of the salinity front is always associated with an El Niño event, but the extremely southward movement events cannot be distinguished from canonical El Niño events. Later we will discuss a climate condition other than El Niño–Southern Oscillation (ENSO) that plays a key role modulating the rainfall pattern and position of the salinity front.

A time series of the possible spawning grounds and the precipitation anomaly may clarify the situation (Fig. 4c). While collective wisdom indicates that Japanese eels always spawn just south of the salinity front in the northwestern Pacific^1^,^2^,^3^,^4^,^6^, the potential spawning grounds can be inferred from the position of the salinity front, which is identified by changes in SSS. The possible spawning ground is indicated by the depth-averaged SSS calculated along 140°E, between 7°N and 18°N. Note that the higher (lower) SSS shows that the possible spawning grounds are located in lower (higher) latitudes, which is unfavorable (favorable) for eel larvae entering the NEC–Kuroshio system. Precipitation anomalies were averaged over the domain between 0–10°N and 130–160°E (black frame in Fig. 4a,b, denoted as P-BOX hereafter), where the greatest rainfall variability occurs in the vicinity of the Japanese eel spawning grounds. The two time series are consistent with each other (γ = −0.5; P < 0.001) (Fig. 4c). The precipitation anomaly in the P-BOX varied among years, with three minima observed during the winters of 1982/83, 1991/92, and 1997/98. Graphically, negative peaks of precipitation anomalies preceded the three SSS maxima by 6 months. The impact of movement of the ITCZ southward across the equator on the Japanese eel spawning grounds is obvious. Applying the climate conditions described above, extreme ITCZ events accompanied by dramatically reduced precipitation in the P-BOX resulted in extreme southward movement of the salinity front and spawning grounds. Thus, the eel larvae failed to join the NEC nursery, which decreased eel catches in East Asian countries.

### Climate phenomena responsible for the ITCZ extremes

We found that variations in movement of the ITCZ were highly correlated with the combination mode (C-mode) index. The C-mode is a previously neglected mode of climate variability generated by the nonlinear interaction between ENSO and the annual cycle in the western Pacific warm pool^12^. This particular mode exhibits characteristic timescales that are clearly distinct from the variability in ENSO. We found a significant correlation between the precipitation anomaly in the P-BOX and the C-mode index (γ = −0.8; P < 0.001) with no time lag during 1980–1999. The relative importance of the C-mode and ENSO can be further quantified in terms of a linear regression model as follows:


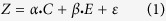


where *Z* represents the precipitation anomaly in the P-BOX, *C* is the C-mode index normalized by its variance, *E* is the Niño 3.4 index normalized by its variance, ε is uncorrelated noise, and α and β are regression coefficients. We found that α = −0.7 and β = −0.4, suggesting that the effect of the C-mode is nearly twice that of ENSO. The result demonstrates that the C-mode rather than ENSO is the dominant climate factor responsible for the precipitation extremes (i.e. cross-equatorial movement of the ITCZ).

**Figure 5:**
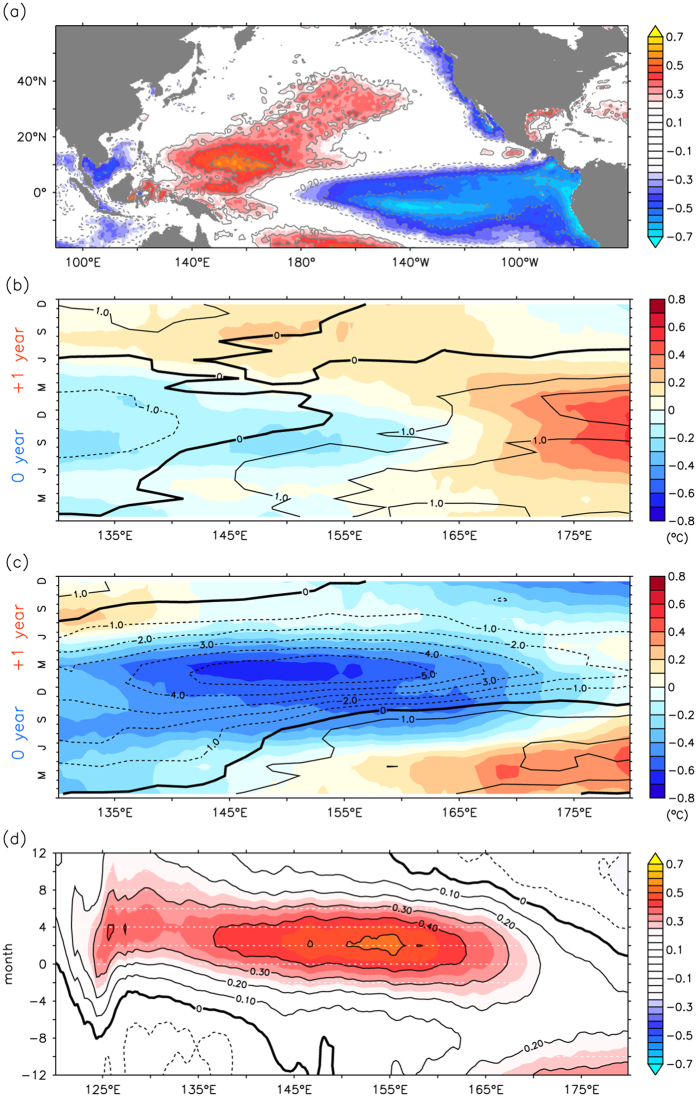
Correlation between precipitation anomaly in the P-BOX and SST anomaly. (**a**) Correlation map (zero lag) between precipitation anomaly in the P-BOX and Pacific SST anomaly from 1982 to 2008. Colored shading indicates statistical significance above 99% confidence level. Contour interval is 0.1. (**b**) SST and precipitation anomaly composite for canonical El Niño events during 1982–2008. Colored shading denotes SST anomaly (unit: °C), while contour is precipitation anomaly (unit: mm/day). Contour interval is 1. (**c**) Same as (**b**), but composite for period of extremely southward movement. (**d**) Lagged correlation between precipitation anomaly in the P-BOX and SST anomaly in the warm pool. Positive month denotes SST changes lead precipitation.

The ITCZ usually develops over the area with the maximum sea surface temperature (SST) in the tropics, where warm water is conducive to enhance deep convection. Thus, SST forcing is largely responsible for the spatial structure of the ITCZ convection^13^. A correlation map was used to examine the relationship between the precipitation anomaly and Pacific SST anomalies from 1982 to 2008 to identify the effect of SST on the ITCZ migration (Fig. 5a). Graphically, there are two significant bands. A positive correlation coefficient (γ > 0.2, 99% significance level = 0.14) is centered at 10°N, 160°E inside the western Pacific warm pool, while another negative correlation coefficient (γ < −0.2) locates in the eastern tropical Pacific where is coincided with the ENSO forcing region. This map indicates that the precipitation anomaly in the P-BOX is highly related to not only SST fluctuations in the warm pool but also the ENSO phenomenon. The warm pool migrates to the southern (northern) hemisphere during winter (summer), inducing cooling (warming) in the low-latitude western North Pacific, which is associated with the annual cycle of solar insolation. The seasonal migration of the warm pool predominates the SST patterns and modulates the rainfall zone in the western tropical Pacific. On the other hand, the ENSO is chiefly responsible for the interannual timescale of SST changes in the area. Figure 5b shows the SST and precipitation anomaly composite for canonical El Niño events during 1982–2008. The warm pool migrates eastward during El Niño, resulting in anomalously warm temperatures in the central and eastern Pacific, but cooling in the western Pacific. This leads to reduced precipitation in the western tropical Pacific. However, ENSO forcing explains only a part of the precipitation pattern. Not only ENSO-driven zonal migration but also sun-driven meridional migration of the warm pool modulate the precipitation pattern and position of the salinity front.

To further investigate the relationship between extremely southward events and SST variability in the warm pool, the composite was performed for the episodes when extreme southward movement occurred (1982/83, 1991/92, and 1997/98). We found dramatically reduced precipitation in the northwestern Pacific (Fig. 5c) that was approximately four-fold more than that of the canonical El Niño shown in Fig. 5b. Furthermore, the significance of SST forcing on ITCZ convection is demonstrated by the lagged correlation between the precipitation anomaly and the SST anomaly in the warm pool (Fig. 5d). A zonal band with a larger correlation coefficient (γ > 0.4) between 137°E and 163°E indicates that SST precedes the precipitation anomaly by 0–4 months. During extreme events, the eastward extension of the warmer water produces anomalously cold SST in the western Pacific, in combination with the warm pool migrating to the southern hemisphere, which leads to strong cooling in the northwestern Pacific. The unique combination of the two is the manifestation of the C-mode, as it is generated by a nonlinear interaction between ENSO and the annual cycle of the warm pool^12^.

**Figure 6:**
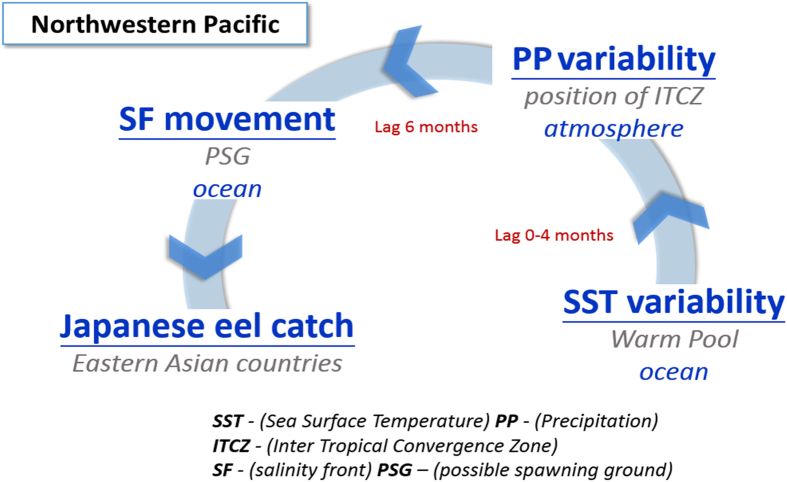
Diagram of the linkage between physical components and the Japanese eel spawning grounds with time lags. A schematic representation of the sequence among the atmospheric/oceanic variability and eel catch in the northwestern Pacific. Significant SST cooling in the warm pool reduces precipitation dramatically, and it precedes the precipitation anomaly (i.e. position of ITCZ) by 0–4 months. Dramatically reduced precipitation results in the salinity maxima or significant southward movement of the salinity front. Furthermore, precipitation anomaly leads the salinity front movement (highly related to the Japanese eel spawning grounds) by 6 months.

The current study shows that the C-mode of climate variability is chiefly responsible for extremely poor recruitment of the Japanese eel. Figure 6 shows diagram of the linkage among climate variabilities and eel catches in East Asia. The SST anomaly in the warm pool precedes the precipitation anomaly (i.e. position of ITCZ) by 0–4 months, while negative peaks in the precipitation anomaly precede the SSS maxima (i.e. extreme southward movement of the salinity front and spawning ground) by 6 months, affecting the Japanese eel catch significantly. As the location of the salinity front is highly related to the potential Japanese eel spawning grounds, either an appropriate SST or precipitation index could serve as a good indicator for eel catches in East Asian countries. Rather than precipitation data, which are generally difficult to obtain and thus are limited, SST images provide practical predictions for the abundance of Japanese eel larvae. One could expect poor recruitment in East Asia 6 months before intense cooling is observed in the northwestern Pacific warm pool.

## Conclusion

This study found that the salinity front shifted farther south in 1983, 1992, and 1998, caused by cross-equatorial movement of the ITCZ. Japanese eels spawned beyond the NEC when the salinity front was extremely southward, and their larvae failed to join the nursery in the current, resulting in a lower eel catch in East Asian countries. Movement of the ITCZ across the equator was closely related to the C-mode rather than ENSO. The eastward extension of warmer water during extreme events produced an anomalously cold SST in the western Pacific in combination with the warm pool, which migrates to the southern hemisphere and leads to strong cooling and dramatically reduced precipitation in the northwestern Pacific.

Furthermore, an appropriate SST index in the western Pacific warm pool is proposed to project the eel catch in East Asian countries. SST images regularly supplied by satellites are capable of predicting the abundance of Japanese eel larvae.

Climate variability aside, other potential factors may also impact on the recruitment success of Japanese eels, such as current fluctuations, ocean eddies^14^, the NEC bifurcation^14^,^15^, available spawner numbers each year, and the larval survival rate^16^. Which of these factors may be important in affecting the decline or interannual recruitment fluctuations of the Japanese eel. Nevertheless, our understanding for this complicated physical/biological system is still murky at the present time. It should become increasingly clear as future samples and data grow in number.

## Methods

### Data sets

a. *Salinity data* are adopted from the Simple Ocean Data Assimilation (SODA, http://apdrc.soest.hawaii.edu/) reanalysis version 2.1.6 with spatial resolution of 0.5° and 40 levels in the vertical. The SODA is driven by winds from the ECMWF Re-Analysis Project (ERA-40) during 1958–2001 and NASA Quick Scatterometer (QuikSCAT) during 2002–2008. Data used for assimilation include hydrographic profiles, moored temperature and salinity time series, and satellite sea surface temperature (SST)^17^.

b. *Precipitation data* are from the Global Precipitation Climatology Project Version 2.2 (GPCP V2.2, http://www.esrl.noaa.gov/psd/data/gridded/data.gpcp.html) monthly precipitation product, which is based on a blend of satellite and *in-situ* measurements since 1979 on global grids (2.5° × 2.5°)^18^.

c. *Sea surface Temperature (SST) data* are obtained from OISST version 2 (http://www.ncdc.noaa.gov/oisst) produced daily on a 0.25° grid at NOAA’s National Climatic Data Center (NCDC). In the present study, we used Daily AVHRR-only OISST product because it has a longer record since 1981.

### El Niño events

Ten El Niño events (1979, 1982, 1986, 1987, 1991, 1994, 1997, 2002, 2004, 2006) during 1979–2007 are included in this study. Canonical El Niño events are 1979, 1986, 1987, 1994. 2002, 2004, 2006, and extreme events are 1982, 1991, and 1997^12^.

### Statistical analyses

Correlation analyses are used to quantify the strength of the relationship between the variables, and the data are based on 5-month running mean (e.g. Fig. 4c). The correlation of significance test is performed based on the two-tailed Student’s t-test.

### Software of Graphics and analyses

All data analyses and figures are generated with FERRET (v6.93; http://ferret.pmel.noaa.gov/Ferret). FERRET is a product of NOAA’s Pacific Marine Environmental Laboratory (PMEL) developed by the Thermal Modeling and Analysis Project (TMAP) at PMEL in Seattle to analyze numerical ocean models’ outputs and compare them with gridded, observational data. FERRET is an interactive computer visualization and analysis environment designed to meet the needs of both oceanographers and meteorologists, and is widely used in the oceanographic community to analyze data and create publication quality graphics.

## Additional Information

**How to cite this article:** Lin, Y.-F. *et al*. A combination mode of climate variability responsible for extremely poor recruitment of the Japanese eel (*Anguilla japonica*). *Sci. Rep.* **7**, 44469; doi: 10.1038/srep44469 (2017).

**Publisher's note:** Springer Nature remains neutral with regard to jurisdictional claims in published maps and institutional affiliations.

## Supplementary Material

Supplementary Information

## Figures and Tables

**Figure 1:**
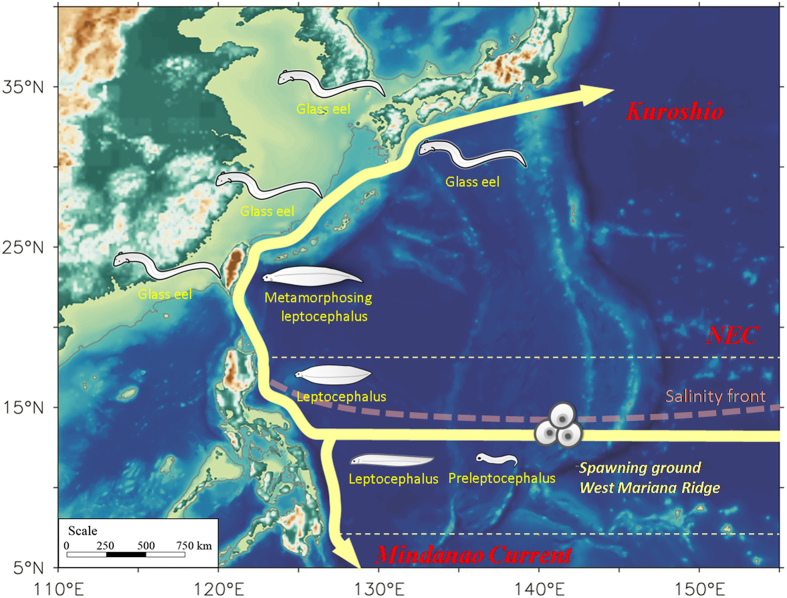
Biogeographic distribution of *A. japonica* in the northwestern Pacific Ocean. Solid arrows are the scheme of circulation pattern for the Kuroshio, North Equatorial Current (NEC), and Mindanao Current. Red dash line denotes position of the salinity front, and yellow dash lines are the range of mean NEC (7–18°N). This figure is modified from Shinoda *et al*.^5^.
