# Supplementary material for: A combination mode of climate variability responsible for extremely poor recruitment of the Japanese eel (Anguilla japonica)
Source: Sci Rep. 2017 Mar 16;7:44469. doi: 10.1038/srep44469 (PMC5353692; doi:10.1038/srep44469)
Supplement: Supplementary Information [file srep44469-s1.pdf]

Supporting Online Material for:

A combination mode of climate variability responsible for extremely poor recruitment of the Japanese eel (Anguilla japonica)

Yong-Fu Lin[1], Chau-Ron Wu[1,*] and Yu-San Han[2]

[1]*Department of Earth Sciences, National Taiwan Normal University, Taipei, Taiwan*
[2]*Institute of Fisheries Science and Department of Life Science, National Taiwan University*

Correspondence to: cwu@ntnu.edu.tw

Observations and tracer experiments

Fig. S1 is annual glass eel catch data for the entire fishing season in East Asia (Taiwan, China, Korea and Japan) during the period from 1995 to 2015. Only the period from 1995 to 2015 is present here because the eel catch data are less reliable before 1995. The figure demonstrates that extremely low eel catch took place only during 1997/98 El Niño.

In situ observations of biological data are limited. There is little direct observational evidence on migration of silver eels to their spawning grounds, and return journeys of the larvae to growth areas in continental waters. Alternatively, the authors design tracer experiments to simulate the possible behavior of the larvae (Fig. S2). The

tracers were released at 100m of depth around 140, 141 and 142°E along the lines of 14°N (normal year) and 12.5°N (extreme year) latitude in July to simulate the main spawning time of *A. japonica*. When tracers were released around 12.5°N during extreme year, they had a much lower probability of entering the Kuroshio than ~14°N during normal year (35.8 % and 42.3 %, respectively). The tracer experiments prove that the latitudinal shift of spawning locations can significantly affect the recruitment success for *A. japonica*, and poor recruitment of the glass eel during extreme year. The results agree with the finding of Kim et al.[1] and Zenimoto et al.[2]

Supplementary Figures:

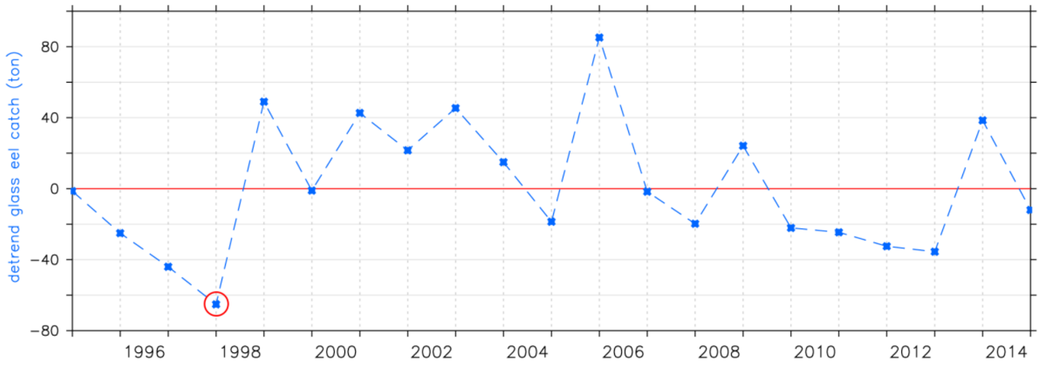

Figure S1. Annual glass eel catch data for the entire fishing season in East Asia (Taiwan, China, Korea, and Japan) during the period from 1995 to 2015.Data were gathered from the Japan Aquaculture Information News (The Nihon Yoshoku Shimbun, Tokyo, Japan).Linear trend has been removed. A significant low eel catch has been found in 1997/98 (red circle). The figure is generated with FERRET (v6.93; http://ferret.pmel.noaa.gov/Ferret).

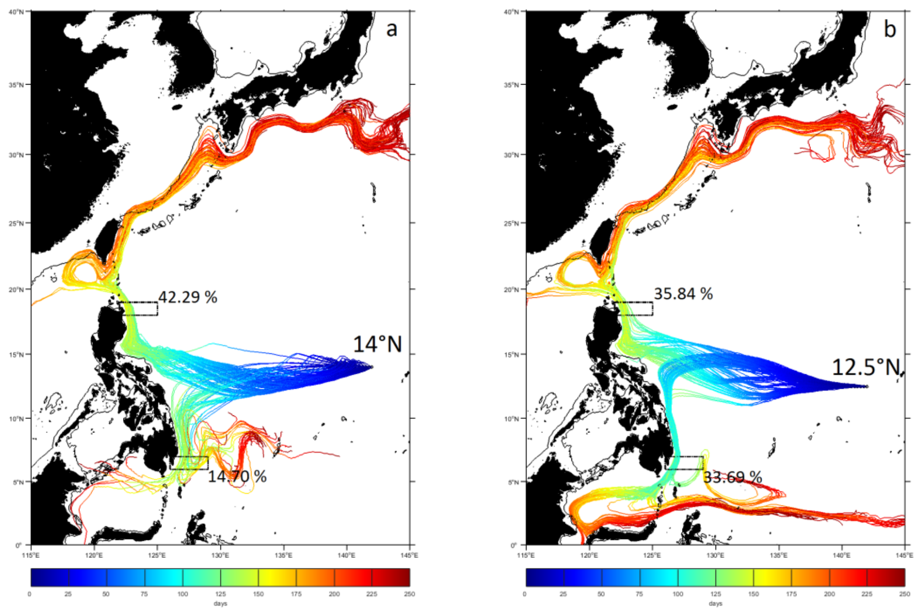

Figure S2. Trajectories from a fixed spawning point of 14°N (a) and 12.5°N (b) associated with meridional displacements of the salinity front in July. Color scale shows cumulative drift days. Percentage of particles that successfully passed the dashed boxes of the Kuroshio (18−19°N and 122−125°E) and Mindanao Current (6−7°N and 126−129°E) was regarded as the successful recruitment rate during the normal and extreme year, respectively. Black contour indicates 200m isobath. All figures are generated with MATLAB (R2015a; http://www.mathworks.com/).

Datasets

a. Glass eel catch data

Annual glass eel catch data for the entire fishing season in East Asia (Taiwan, China, Korea, and Japan) during the period from 1995 to 2015.Data were gathered from the Japan Aquaculture Information News (The Nihon Yoshoku Shimbun, Tokyo, Japan).

b. North Pacific Ocean model

The study used 20-year (1990~2009) current velocity data from the North Pacific Ocean (NPO) model[3,4] based on the Princeton Ocean Model (POM) (Blumberg & Mellor 1987). The NPO model is highly consistent with Archiving, Validation and Interpretation of Satellite Oceanographic (AVISO) data and observation data (e.g. shipboard acoustic Doppler current profilers, Sb-ADCP)[5]. Based on hydrostatic approximate analysis, this model solves 3-dimensional primitive equations for momentum, salinity, and heat, and estimates turbulence by the Mellor-Yamada level 2.5 scheme[3]. The NPO model covers the domain of the northern Pacific Ocean from 30°S to 65° N and 99° E to 71°W, with a horizontal resolution of 0.25 × 0.25° and 26 sigma levels in the vertical. The model is driven by the monthly 2.5 × 2.5° NCEP-DOE 10m wind climatology derived from averaging all historical data month by month (http://www.esrl.noaa.gov/psd/data). In addition, the monthly climatology of the Simple Ocean Data Assimilation (SODA) reanalysis products, similarly derived from

averaging over all available years with 0.5° resolution (http://apdrc.soest. hawaii.edu/), provides lateral boundary conditions for the NPO model. After spinning up, the NPO model is subsequently forced by 6-hourly NCEP-DOE reanalysis surface winds from 1948 to 2008. Furthermore, the monthly SODA data provide the open boundary conditions for the NPO model[4,6,7].

Tracer experiments

The NPO model is a credible model for tracer experiments[4,6], and was used here to understand the presumed passive behavior of eel larvae. Because the spawning area of *Anguilla japonica* was located latitudinally between 12 and 15°N within the constant westward flow of the NEC, and longitudinally along the western side of the West Mariana Ridge[8], the larvae dispersal patterns of *Anguilla japonica* were thus simulated at 100m depth, starting around 14 (normal year) and 12.5°N (extreme year), respectively. The float particles were released at 140, 140.25, 140.50, 140.75, 141, 141.25, 141.50, 141.75, and 142°E from July 1 to July 31. Thus, each simulation cast float particles (9 particles × 31 days). The percentage of the particles that successfully passed the dashed boxes of the Kuroshio (18−19°N and 122−125°E) and Mindanao Current (6−7°N and 126−129°E) was regarded as the rate of successful recruitment.

Reference

1. Kim, H., Kimura, S., Shinoda, A., Kitagawa, T., Sasai, Y., & Sasaki, H. Effect of

El Niño on migration and larval transport of the Japanese eel (*Anguilla japonica*). *ICES J. Mar. Sci.*, **64**, 1387-1395 (2007).

2. Zenimoto, K., Kitagawa, T., Miyazaki, S., Sasai, Y., Sasaki, H., & Kimura, S. The effects of seasonal and interannual variability of oceanic structure in the western Pacific North Equatorial Current on larval transport of the Japanese eel *Anguilla japonica*. *J. Fish Biol.*, **74**, 1878-1890 (2009).
3. Hsin, Y.-C., Wu, C.-R., & Shaw, P.-T. Spatial and temporal variations of the Kuroshio east of Taiwan, 1982-2005: A numerical study.*J. Geophys. Res. Oceans*, **113**, C04002 (2008).
4. Hsin, Y.-C., Wu, C.-R., & Chao, S.-Y. An updated examination of the Luzon Strait transport. *J. Geophys. Res.*, **117**, C03022 (2012).
5. Hsin, Y.-C., Qiu, B., Chiang, T.-L., &Wu, C.-R.Seasonal to interannual variations in the intensity and central position of the surface Kuroshio east of Taiwan. *J. Geophys. Res. Oceans*, **118**, 4305-4316 (2013).
6. He X., Bai Y., Chen, C.-T. A., Hsin, Y.-C., Wu, C.-R., W. Zhai, Liu, Z., & Gong, F. Satellite views of the episodic terrestrial material transport to the southern Okinawa Trough driven by typhoon. *J. Geophys. Res.*, **119**, 4490−4504 (2014).
7. Han, Y.-S., Lin, Y.-F., Wu,C.-R., Iizuka, Y., Castillo, T., Yambot, I., Mamalangkap, M., & Yambot, A.V. Biogeographic distribution of the eel *Anguilla luzonensis*:

dependence upon larval duration and oceanic currents.*Mar. Ecol. Prog. Ser.*, **551**, 227-238 (2016).

8. Aoyama, J., Watanabe, S., Miller, M. J., Mochioka, N., Otake, T., Yoshinaga, T., & Tsukamoto, K. Spawning sites of the Japanese eel in relation to oceanographic structure and the West Mariana Ridge. *PLoS ONE*, **9**, e88759 (2014).
